# Using sleep heart rate variability to investigate the sleep quality in children with obstructive sleep apnea

**DOI:** 10.3389/fpubh.2023.1103085

**Published:** 2023-02-27

**Authors:** Li-Ang Lee, Hai-Hua Chuang, Hui-Shan Hsieh, Chao-Yung Wang, Li-Pang Chuang, Hsueh-Yu Li, Tuan-Jen Fang, Yu-Shu Huang, Guo-She Lee, Albert C. Yang, Terry B. J. Kuo, Cheryl C. H. Yang

**Affiliations:** ^1^Department of Otorhinolaryngology–Head and Neck Surgery, Linkou Chang Gung Memorial Hospital, Taoyuan, Taiwan; ^2^Faculty of Medicine, Graduate Institute of Clinical Medicine Sciences, Chang Gung University, Taoyuan, Taiwan; ^3^Sleep Center, Metabolism and Obesity Institute, Linkou Chang Gung Memorial Hospital, Taoyuan, Taiwan; ^4^School of Medicine, National Tsing Hua University, Hsinchu, Taiwan; ^5^Institute of Brain Science, National Yang Ming Chiao Tung University, Taipei CIty, Taiwan; ^6^Department of Family Medicine, Linkou Chang Gung Memorial Hospital, Taoyuan, Taiwan; ^7^Department of Otolaryngology, Xiamen Chang Gung Hospital, Xiamen, Fujian, China; ^8^Department of Cardiology, Linkou Chang Gung Memorial Hospital, Taoyuan, Taiwan; ^9^Department of Pulmonary and Critical Care Medicine, Linkou Chang Gung Memorial Hospital, Taoyuan, Taiwan; ^10^Department of Child Psychiatry, Linkou Chang-Gung Memorial Hospital, Taoyuan, Taiwan; ^11^Department of Otolaryngology, Taipei City Hospital, Taipei City, Taiwan; ^12^Department of Psychiatry, Taipei Veterans General Hospital, Taipei City, Taiwan; ^13^Center for Mind and Brain Medicine, Tsaotun Psychiatric Center, Ministry of Health and Welfare, Nantou City, Taiwan; ^14^Sleep Research Center, National Yang Ming Chiao Tung University, Taipei City, Taiwan

**Keywords:** adenotonsillectomy, children, heart rate variability (HRV), mediation, obstructive sleep apnea, quality of life

## Abstract

**Background:**

Obstructive sleep apnea (OSA) is associated with impaired sleep quality and autonomic dysfunction. Adenotonsillectomy significantly improves subjective and objective sleep quality in children with OSA. However, the postoperative changes in heart rate variability (HRV) indices (indicators of cardiac autonomic function) and their importance remain inconclusive in childhood OSA. This retrospective case series aimed to investigate the association of sleep HRV indices, total OSA-18 questionnaire score (a subjective indicator of sleep quality) and polysomnographic parameters (objective indicators of sleep quality), and effects of adenotonsillectomy on HRV indices, total OSA-18 questionnaire score and polysomnographic parameters in children with OSA.

**Methods:**

Seventy-six children with OSA were included in baseline analysis, of whom 64 (84%) completed at least 3 months follow-up examinations after adenotonsillectomy and were included in outcome analysis. Associations between baseline variables, and relationships with treatment-related changes were examined.

**Results:**

Multivariable linear regression models in the baseline analysis revealed independent relationships between tonsil size and obstructive apnea-hypopnea index (OAHI), adenoidal-nasopharyngeal ratio and very low frequency (VLF) power of HRV (an indicator of sympathetic activity), and normalized low frequency power (an indicator of sympathetic activity) and OAHI. The outcome analysis showed that adenotonsillectomy significantly improved standard deviation of all normal-to-normal intervals, and high frequency power, QoL (in terms of reduced total OSA-18 questionnaire score), OAHI and hypoxemia. Using a conceptual serial multiple mediation model, % change in OSA-18 questionnaire score and % change in VLF power serially mediated the relationships between change in tonsil size and % change in OAHI.

**Conclusions:**

The improvement in OAHI after adenotonsillectomy was serially mediated by reductions in total OSA-18 questionnaire score and VLF power. These preliminary findings are novel and provide a direction for future research to investigate the effects of VLF power-guided interventions on childhood OSA.

## 1. Introduction

Over 4% of children worldwide suffer from obstructive sleep apnea (OSA) (1). OSA, characterized by snoring and abnormal breathing during sleep, is a chronic disorder with many comorbidities, including cardiovascular sequelae (2) and cognitive/behavioral problems (3). OSA considerably reduces sleep quality in children (4). Furthermore, childhood OSA has been associated with hypofunction in brain autonomic control regions (5), which can influence heart rate and heart rate variability (HRV) by the interposition of cortico-subcortical pathways to the sympathetic nervous system (SNS) and parasympathetic nervous system (PNS) (6).

Unlike clinical signs and symptoms, which are often direct presentations of a disease, HRV reflects more indirect underlying pathophysiological process, either causal, mediating, or reactive, which allows measurements of the HRV to serve as a biomarker in a wide range of health conditions (7). Time domain and frequency domain HRV analysis on electrocardiograms are useful for diagnosing different clinical and functional conditions (8). For example, 24-h HRV indices are significantly associated with sleep disturbance and depression symptoms of medical students (9). In children with OSA, sleep fragmentation, arousal, and hypoxemia may increase SNS activity (10). However, sleep stage-specific HRV measurements have shown significantly downregulated PNS activity in children with sleep-disordered breathing (11). Studies on HRV in children with OSA have reported inconsistent results (12–14), and thus further investigations on cardiac autonomic function in this population are warranted.

Hypertrophy of adenoids and tonsils is the most common cause of upper airway obstruction in children (15), and adenotonsillectomy is the first-line treatment for childhood OSA (12, 16). Adenotonsillectomy significantly reduces the severity of OSA in terms of apnea-hypopnea index (AHI) and sympathetic activity (17) and sustainably improved quality of life (18). However, approximately 70% of children have residual OSA (19), which still threatens children's health. Further, changes in OSA-related HRV indices are not related to changes in AHI and hypoxemia (14). Accordingly, the aims of this study were to evaluate the reproducibility of sleep HRV analysis, the associations of sleep HRV and sleep quality, and the changes in HRV indices after adenotonsillectomy in children with OSA, and understand how these changes relate to adenoid-tonsil size and improvements in polysomnographic parameters.

## 2. Materials and methods

### 2.1. Study participants

The Institutional Review Board of Chang Gung Medical Foundation approved this retrospective case series (No. 202200882B0). The requirement for written informed consent was waived because the current study was based on a secondary analysis of existing data. This study followed the World Medical Association's Declaration of Helsinki and the Strengthening the Reporting of Cohort Studies in Surgery guidelines (20).

**Figure 1:**
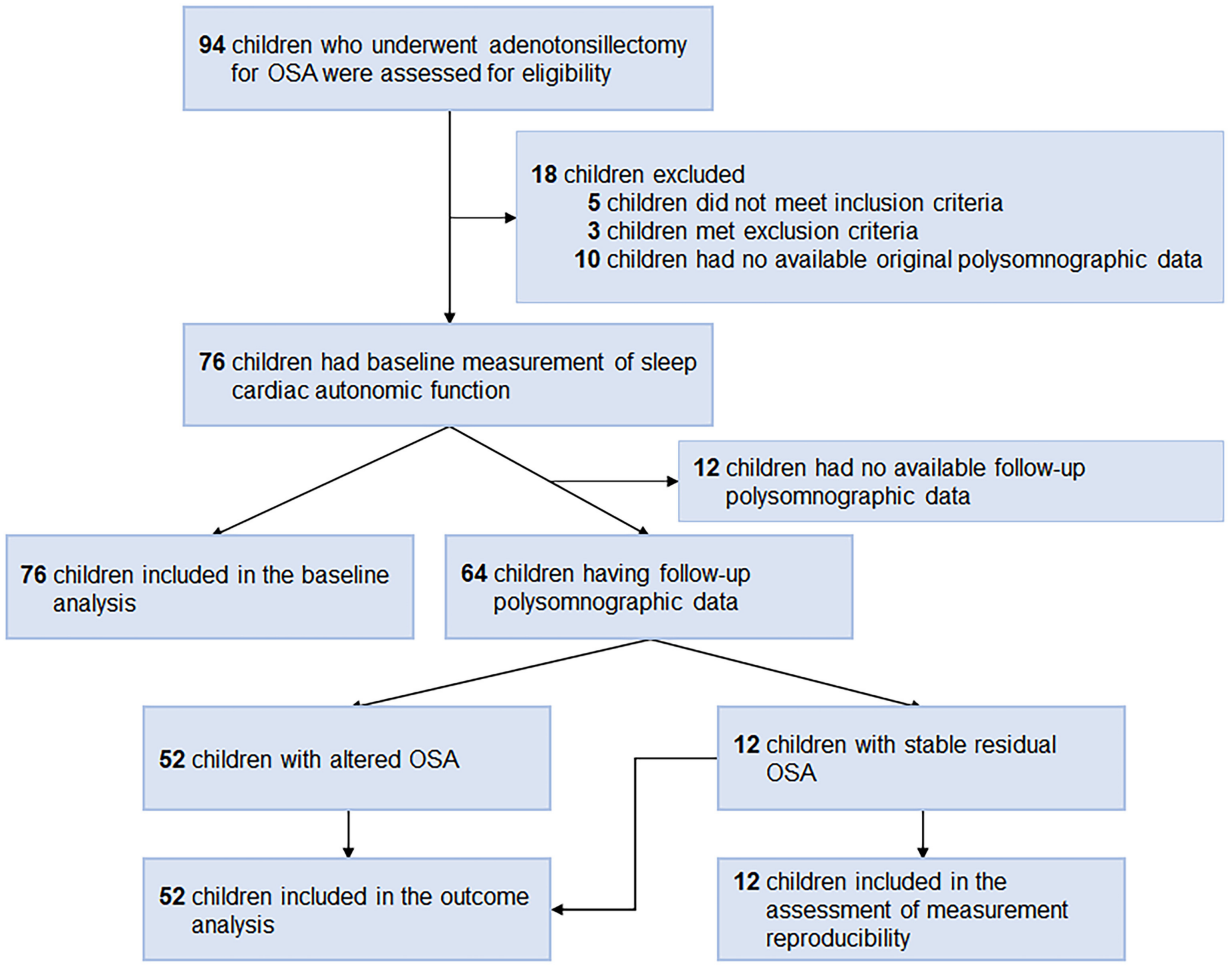
Flow diagram. OSA, obstructive sleep apnea.

We included consecutive children who underwent adenotonsillectomy for OSA at Chang Gung Memorial Hospital, Linkou Main Branch (Taoyuan, Taiwan) between March 1, 2017 and September 30, 2021. The inclusion criteria were: (1) age 5–12 years, and (2) obstructive AHI (OAHI) ≥ 2.0 events/h or obstructive apnea index (OAI) ≥ 1.0 events/h (21, 22). The exclusion criteria were (1) patients with craniofacial, neuromuscular, or chronic inflammatory disorders (23, 24), or (2) patients without available polysomnographic data. All the children underwent extracapsular tonsillectomy with tonsillar pillar suturing and adenoidectomy that aimed to improve the upper airway obstruction by the principal investigator (L-AL) in a single stage under general anesthesia (25). Children with follow-up polysomnographic data were included in outcome analysis (Figure 1).

### 2.2. Clinical variables

Age, sex, body mass index (BMI), tonsil size, adenoidal-nasopharyngeal ratio (ANR) and evening blood pressure (BP) (2, 26), OSA-related quality of life, and polysomnographic parameters were recorded. All the clinical measurements were performed before and at least 3 months after adenotonsillectomy.

The tonsils were graded with a size scale from 1–4 (1: tonsils within the tonsillar; 2: tonsils visible outside the anterior pillars; 3: tonsils extending three-quarters of the way to the midline; 4: tonsils meeting at the midline) (27).

The ANR (distance from the point of maximal convexity of the adenoid shadow/the distance between the posterior border of the hard palate and the anteroinferior edge of the sphenobasioccipital synchondrosis) was measured on neck lateral view (28).

### 2.3. Sleep quality

#### 2.3.1. Subjective measurement

All parents evaluated their children's OSA-related quality of life using the Chinese version of the OSA-18 questionnaire (29), which includes 18 items grouped into 5 domains: sleep disturbance (4 items), physical suffering (4 items), emotional distress (3 items), daytime problems (3 items), and caregiver concerns (4 items). Each item was scored using a 7-point ordinal scale. The total score was calculated as the sum of the 18 items (overall range, 18–126) and has been shown to have excellent test-retest reliability (30).

#### 2.3.2. Objective measurement

All participants underwent full-night, in-laboratory polysomnography (Nicolet Biomedical Inc., Madison, WI, USA) (23). OAHI, OAI, arousal index, mean blood oxygen saturation (SaO_2_), minimal SaO_2_, sleep stages and total sleep time were scored and manually verified by the study investigators (L-PC and Y-SH) using a standard approach of the American Academy of Sleep Medicine (31). For example, the AHI was calculated by dividing the sum of all apneas (defined as a ≥ 90% reduction in airflow for a duration of ≥ 2 consecutive breaths) and hypopneas (defined as a ≥ 30% reduction in airflow in association with electroencephalographic arousal or a ≥ 3% reduction in SpO_2_ for a duration of ≥ 2 consecutive breaths) by the hours of total sleep time.

### 2.4. Sleep heart rate variability analysis

Electrocardiographic polysomnography signals were analyzed using HRV software (profusionSLEEP^TM^, version 4.5, build 502, Compumedics, Abbotsford, Australia). For artifact correction, automated annotations of electrocardiographic signals, such as loose leads, motion artifacts, and broken wires (32), were manually verified by trained technicians who had been certificated by the domestic board of the Taiwan Society of Sleep Medicine and shown substantial-to-almost perfect reliabilities in the scoring of respiratory events (intraclass correlation coefficients [ICCs] ranged from 0.66 to 0.98) (33). According to standard guidelines, time-domain indices, including standard deviation of all normal-to-normal (N-N) intervals (SDNN), number of pairs of adjacent N-N intervals differing by more than 50 ms in the entire recording divided by the total number of all N-N intervals (pNN50), and square root of the mean of the sum of the squares of differences between adjacent N-N intervals (RMSSD) were recorded. In addition, frequency-domain indices, including total power (0.0033–0.4 Hz), very low frequency (VLF) power (0.0033–0.04 Hz), low frequency (LF) power (0.04–0.15 Hz), normalized LF power (LF%), high frequency (HF) power (0.15–0.4 Hz), and LF/HF ratio were also recorded (Table 1) (34–39).

**Table 1:** Indices, units, descriptions, and meanings of heart rate variability.

**Variable**	**Unit**	**Description**	**Meaning**
**Time-domain indices**			
N-N	ms	Time interval between N-N heartbeats	
SDNN	ms	Standard deviation of all N-N intervals.	Total capacity of the regulation system (35)
pNN50	%	Number of pairs of adjacent N-N intervals differing by more than 50 ms divided by the total number of all N-N intervals.	Increased parasympathetic activity (35)
RMSSD	ms	The square root of the mean of the sum of the squares of di?erences between adjacent N-N intervals.	Increased parasympathetic activity (36)
**Frequency-domain indices**			
Total power	ms^2^	The variance of N-N intervals over the approximately the temporal segment (approximately ≤ 0.4 Hz)	Total capacity of the regulation system (35)
VLF power	ms^2^	Power in very low frequency range ( ≤ 0.04 Hz)	Sympathetic activity (36)
LF power	ms^2^	Power in low frequency range (0.04–0.15 Hz)	Baroreceptor activity (37)
LF%	%	LF power / (Total Power–VLF power) × 100	Sympathetic modulation (38)
HF power	ms^2^	Power in high frequency range (0.15–0.4 Hz)	Parasympathetic modulation (39)
LF/HF ratio		Ratio LF [ms^2^]/HF [ms^2^]	Sympathovagal balance (34)

HF, high frequency; LF, low frequency; LF%, normalized LF power; N-N, normal-to-normal; pNN50, proportion of N-N50 divided by the total number of N-N intervals; RMSSD, square root of the mean of the sum of the squares of di?erences between adjacent N-N intervals; SDNN, standard deviation of all N-N intervals; VLF, very low frequency.

### 2.5. Reproducibility assessment

Reproducibility of the HRV measurements was assessed using ICCs (two-way random model; absolute agreement type) from data quantified from separate sleep HRV measurements performed at least 3 months apart in a sample of 12 children with stable residual OSA [defined as postoperative OAHI within (preoperative OAHI−5.6 events/h) to (preoperative OAHI + 6.8 events/h), compatible with the upper and lower limits of agreement of OAHI measured on the first and second night in children and adolescents] (40). This sample represented children who did not undergo adenotonsillectomy. ICCs evaluated reproducibility as “poor” (< 0.001), “slight” (0.001–0.020), “fair” (0.021–0.40), “moderate” (0.41–0.60), “substantial” (0.61–0.80), and almost perfect (0.81–1.00) (41).

### 2.6. Statistical analysis

Data were analyzed using SPSS version 25.0 (IBM Corp., Armonk, NY, USA) and GraphPad Prism 9.0 for Windows (Graph Pad Software Inc., San Diego, CA, USA). Changes in scores were calculated as postoperative minus preoperative values. Percentage change [(change in score/preoperative value) × 100] was calculated for variables of interest. Because all the children underwent extracapsular tonsillectomy, the change in tonsil size was equal to the negative value of tonsil size and used for further statistical analysis.

Using the Shapiro-Wilk test to examine normality, descriptive statistics were expressed as mean (standard deviation) for normally distributed continuous variables, median (interquartile range [IQR]) for skewed variables, and number (proportion) for categorical variables.

For continuous variables, the independent-samples *t*-test or Mann-Whitney *U* test was used to assess between-group changes; the paired-samples *t*-test or Wilcoxon signed-rank test was used to assess within-group changes as appropriate. Differences in categorical variables between two subgroups were analyzed using Fisher's exact test.

To facilitate comparisons with previous studies, linear regression models, or mediation and moderation analysis, non-normally distributed data of reference studies were transformed to normal after estimation from the sample size (*n*), median (*m*), and the first (*q*_1_) and third (*q*_3_) quartiles (42, 43). The sample standard deviation was estimated to be [(*q*_3_ – *q*_1_) / η] where η = η(*n*) = 2Φ^−1^[(0.75 × *n* – 0.125) / (*n* + 0.25)] (42). In addition, non-normally distributed continuous variables were transformed to normal using a two-step approach: fractional rank and inverse-normal transformation (44). For comparisons with reference values, the one-sample *t*-test was applied.

Relationships between variables of interest were assessed using Pearson and Point-Biserial correlation tests as appropriate. Multivariable linear regression models, including all variables, with manual selection based on a probability of F < 0.05 were used to identify independent variables. The variance inflation factor of each predictor was calculated to adjust for intervariable relationships within the model. The regression model was repeated after removing all variables with a variance inflation factor ≥ 5 to reduce multicollinearity (45).

Conditional process analysis was performed to evaluate the mediators and moderators between changes in tonsil size/ANR and % changes in polysomnographic parameters using the SPSS PROCESS macro (version 4.1) (46). Bias-corrected 95% confidence intervals (CIs) were estimated *via* bootstrapping (5,000 runs) to verify mediation, moderated mediation, or mediated moderation. A two-sided *P* < 0.05 was considered statistically significant.

## 3. Results

### 3.1. Participants' characteristics

Seventeen (22%) girls and 59 (78%) boys with OSA (median OAHI, 5.5 [IQR, 2.3–12.6] events/h) were included in the baseline analysis (Figure 1), of whom 64 (84%) were included in the outcome analysis and 12 (16%) were not included due to no available follow-up polysomnography. All baseline variables were comparable between these two subgroups (Table 2).

**Table 2:** Demographics of the participants in the baseline analysis and those included and excluded from the outcome analysis.

**Variable**	**Participants included in the baseline analysis**	**Participants included in the outcome analysis**	**Participants excluded in the outcome analysis**	***P* value^a^**
***N***	**76**	**64**	**12**	
**Clinical variables**				
Age at diagnosis (years)	7 (6–9)	6 (5–9)	7 (6–10)	0.417
Male sex, *n* (%)	59 [78]	47 [73]	12 (100)	0.058
BMI (kg/m^2^)	17.4 (15.3–22.8)	17.3 (15.0–21.9)	21.1 (15.6–26.8)	0.133
Tonsil size	3 (3–4)	3 (3–4)	3 (3–4)	0.703
ANR	0.800 (0.697–0.872)	0.800 (0.710–0.864)	0.691 (0.585–0.848)	0.093
Systolic BP (mmHg)	104.2 (18.0)	103.5 (17.2)	107.9 (22.1)	0.440
Diastolic BP (mmHg)	65 (59–71)	65 (59–71)	66 (57–76)	0.825
**Subjective sleep quality (assessed by the OSA-18 questionnaire)**				
OSA-18 score	81.3 (15.4)	81.8 (15.7)	78.4 (14.1)	0.486
**Objective sleep quality (assessed by polysomnography)**				
OAHI (events/h)	5.5 (2.3–12.6)	5.8 (2.4–13.1)	6.3 (2.4–10.4)	0.943
OAI (events/h)	0.4 (0.1–1.5)	0.6 (0.2–1.7)	0.5 (0.18–1.5)	0.908
Arousal index (events/h)	9.8 (7.2–16.5)	9.9 (7.3–16.9)	9.0 (6.7–15.2)	0.397
Mean SaO_2_ (%)	97 (97–98)	97 (97–98)	97 (96–98)	0.480
Minimal SaO_2_ (%)	84 (90–92)	90 (84–92)	90 (83–92)	0.797
N1 sleep (%)	10 (6–15)	10 (6–16)	9 (7–14)	0.569
N2 sleep (%)	39.1 (8.6)	39.2 (9.2)	39.0 (4.8)	0.911
N3 sleep (%)	28 (22–35)	27 (22–35)	29 (23–35)	0.711
REM sleep (%)	19.3 (5.9)	19.1 (5.8)	20.2 (6.3)	0.556
TST (min)	337 (321–352)	337 (320–353)	329 (321–349)	0.437
**Sleep heart rate variability indices**				
Heart rate (bpm)	76 (70–82)	76 (70–85)	76 (70–82)	0.770
N-N interval (ms)	791.8 (97.3)	793.7 (95.6)	781.6 (93.3)	0.694
SDNN (ms)	96.6 (32.6)	98.3 (34.1)	87.4 (21.7)	0.163
pNN50 (%)	36.9 (19.0)	37.1 (19.2)	35.8 (5.5)	0.840
RMSSD (ms)	67 (50–105)	63 (49–114)	69 (53–80)	0.680
Total power (ms^2^)	8688 (4614–14944)	9454 (4499–15430)	7560 (5233–9443)	0.298
VLF power (ms^2^)	1509 (1095–2540)	1509 (1095–2727)	1479 (895–1833)	0.340
LF power (ms^2^)	1236 (760–2150)	1243 (734–2507)	992 (804–1526)	0.243
LF% (%)	37 (28–47)	38 (29–48)	33 (27–42)	0.494
HF power (ms^2^)	2140 (1113–4228)	1970 (1103–5155)	2183 (1248–4412)	0.669
LF/HF ratio	0.59 (0.40–0.90)	0.62 (0.40–0.92)	0.50 (0.40–0.70)	0.459

Data are expressed as mean (standard deviation), median (interquartile range), or number (%).

^a^Data were compared between participants included in the outcome analysis and those excluded using the independent-samples t-test, Mann-Whitney U test, or Fisher's exact test as appropriate.

ANR, adenoidal-nasopharyngeal ratio; BMI, body mass index; BP, blood pressure; bpm, beats per min; HF, high frequency; LF, low frequency; LF%, normalized LF power; N-N, normal-to-normal; OAHI, obstructive apnea-hypopnea index; OAI, obstructive apnea index; OSA, obstructive sleep apnea; pNN50, proportion of N-N50 divided by the total number of N-N intervals; REM, rapid eye movement; RMSSD, square root of the mean of the sum of the squares of di?erences between adjacent N-N intervals; SaO_2_, blood oxygen saturation; SDNN, standard deviation of all N-N intervals; TST, total sleep time; VLF, very low frequency.

### 3.2. Sleep heart rate variability

**Figure 2:**
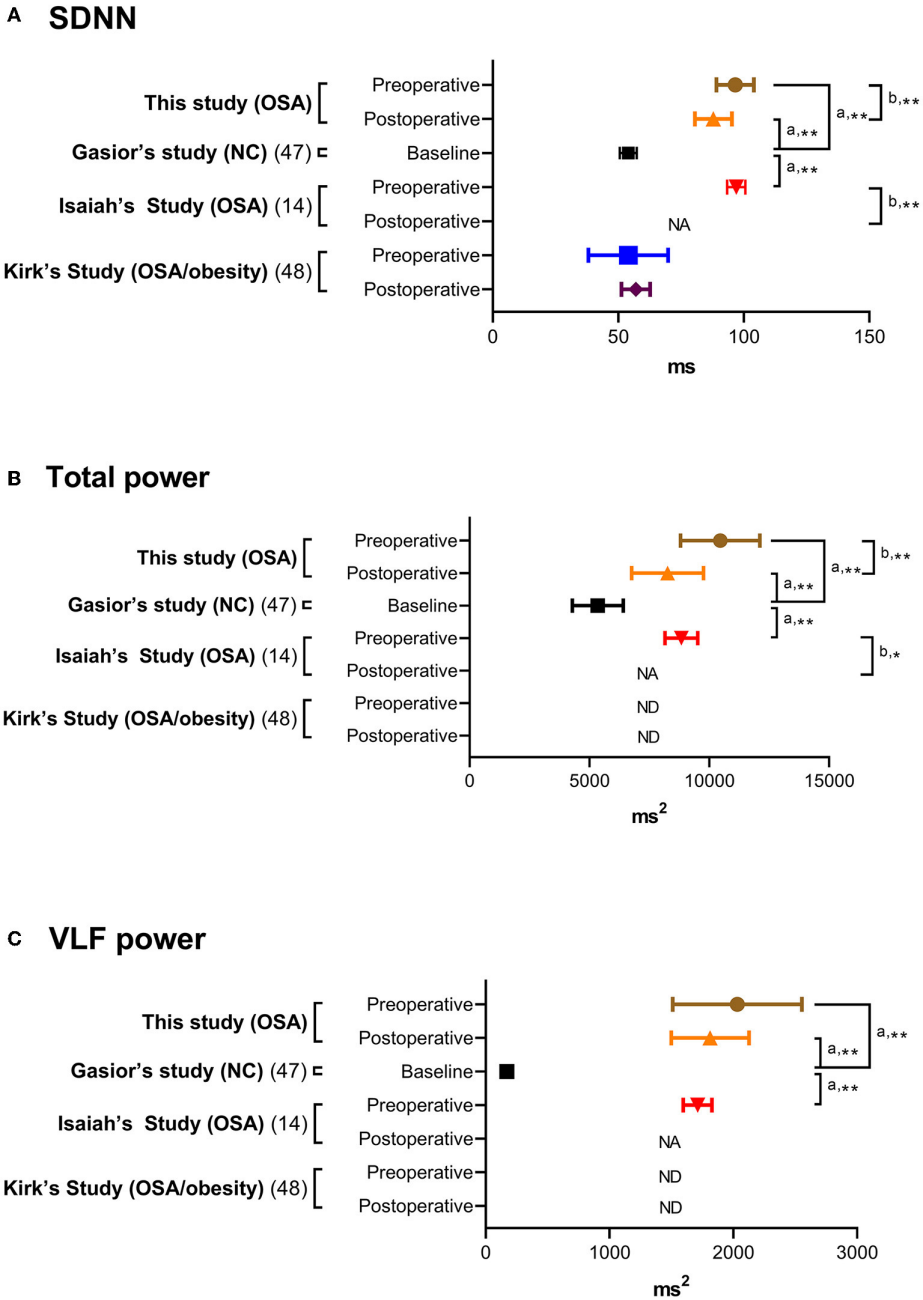
**(A–C)** Full-night heart rate variability indices in normal controls and children and adolescents with obstructive sleep apnea. Data are summarized as means and 95% confidence intervals. ^a^The one-sample *t*-test was applied for comparisons of related samples according to the reference values of normal control (47). ^b^The paired-samples *t*-test or Wilcoxon signed-rank test was used to compare related samples according to the original references (14, 48). ^*^P < 0.05 and ≥0.01; ^**^P < 0.01 and ≥0.001. NA, not available; ND, not detected; OSA, obstructive sleep apnea; SDNN, standard deviation of all normal-normal intervals; VLF, very low frequency.

Distributions of HRV indices in baseline analysis are summarized in Table 2. For comparing with previous studies, the HRV indices in this study (full-night), normal controls (full-night) (47), children with OSA (full-night) (14), children with moderate-to-severe OSA (N3 sleep) (13, 17), and children with OSA/obesity (full-night) (48) are summarized in Table 3. Comparing with three representative full-night HRV studies (14, 47, 48), SDNN, total power and VLF power in the children with OSA were significantly higher than normal values (Figure 2).

**Table 3:** Sleep heart rate variability indices in normal controls and in children and adolescents with OSA.

**Variable**	**Our study**	**Gasior's study (47)**	**Isaiah's study (14)**	**Muzumdar's study** **(****17****)**			**Nisbet's study** **(****13****)**						**Kirk's Study (48)**
Publication year		2020	2020	2011			2013						2020
Nation	Taiwan	Poland	USA	USA			Australia						Canada
Participants	OSA	NC	OSA	M-S OSA			Mild OSA			M-S OSA			OSA/obesity
Case number	76	312	404	18			39			29			12
Age (years)	7.2 (2.1)	10.1 (2.5)	6.0 (4.5)	4.9 (2.4)			4.3 (0.1)			4.2 (0.2)			12.8 (5.1)
Male sex, *n* (%)	59 (78)	159 (51)	195 (48)	13 (72)			26 (67)			18 (62)			10 (83)
OAHI (events/h)	10.2 (12.6)	NA	4.6 (4.6)^*^	31.9 (24.8)^*^			3.1 (0.9)			15.5 (12.3)			13.8 (14.5)
Sleep stage	FN	FN	FN	N1, N2	N3	REM	N1, N2	N3	REM	N1, N2	N3	REM	FN
Heart rate (bpm)	77 (9)	NA	NA	100 (17)^a^	100 (15)^a^	107 (16)^a^	NA			NA			82 (7)
N-N interval (ms)	792 (97)	NA	NA	630 (120)^b^	620 (100)^b^	580 (110)^a^	NA			NA			732 (62)
SDNN (ms)	97 (33)	54 (30)	97 (37)	NA			NA			NA			54 (25)
pNN50 (%)	37 (19)	34 (52)	36 (24)	NA			NA			NA			NA
RMSSD (ms)	76 (38)	71 (91)	81 (45)	61 (55)^b^	43 (29)^b^	76 (57)^b^	NA			NA			NA
Total power (ms^2^)	10,460 (7,238)	5,348 (9,534)	8,838 (6,990)	NA			7,632 (5,908)	6,021 (6,145)	5,287 (4,103)	9,040 (5,905)^a^	6,600 (6,144)	5,827 (4,789)^a^	NA
VLF power (ms^2^)	2,032 (2,284)	171 (284)	1,712 (1,186)^a^	NA			NA			NA			NA
LF power (ms^2^)	1,727 (1,457)	2,023 (3,789)	1,382 (1,186)	360 (342)	184 (148)^b^	307 (293)	1,298 (1,024)	758 (781)	909 (606)	1,959 (1,023)^a^	848 (780)	1,075 (603)^a^	NA
LF% (%)	39 (15)	NA	NA	NA			NA			NA			41 (17)
HF power (ms^2^)	3,149 (3,029)	3,766 (6,993)	2,742 (3,340)	902 (1,202)^b^	483 (497)^b^	412 (449)^b^	4,418 (3,610)^a^	3,985 (3,859)	2,416 (2,186)^a^	7,382 (3,608)^a^	4,126 (3,856)	2,183 (2,186)	NA
LF/HF ratio	0.80 (0.66)	0.72 (0.82)	0.53 (0.36)	1.60 (2.7)^a^	1.20 (1.60)^a^	3.0 (5.4)^a^	0.70 (0.62)	0.40 (0.25)	1.10 (0.62)	0.70 (0.54)	0.30 (0.27)^b^	0.90 (0.54)	1.40 (1.18)

To facilitate comparisons with previous studies, data are summarized as mean (standard deviation) after estimation from the sample size, median, and interquartile range.

^a^Higher than controls, *P* < 0.05; ^b^Lower than controls, *P* < 0.05.

^*^Only AHI was available.

AHI, apnea-hypopnea index; bpm, beats per min; FN, full night; HF, high frequency; LF, low frequency; LF%, normalized LF power; M-S, moderate-to-severe; N3, stage 3 sleep; NC, normal controls; N-N, normal-to-normal; pNN50, proportion of N-N50 divided by the total number of N-N intervals; RMSSD, square root of the mean of the sum of the squares of differences between adjacent N-N intervals; SDNN, standard deviation of all N-N intervals; VLF, very low frequency.

### 3.3. Measurement reproducibility of sleep heart rate variability

To assess measurement reproducibility, we calculated ICCs using HRV indices measured at least 3 months apart in 12 patients with stable residual OSA after adenotonsillectomy (Table 4). Their variables of interest were comparable to the patients with altered OSA (Tables 5, 6). Most HRV measurements demonstrated moderate (N-N interval, SDNN, RMSSD, total power, LF%) or substantial (VLF power, LF power, LF/HF ratio) reproducibility. Further, the reproducibility of pNN50 and HF power were fair (41).

**Table 4:** Reproducibility of sleep HRV measurements in twelve children with stable residual OSA after adenotonsillectomy.

**Variable**	**Preoperative**	**Postoperative**	**ICC**	**95% CI**	***P*-value**
**Time-domain indices**					
N-N interval (ms)	803 (685–870)	829 (728–855)	0.488	−0.123–0.823	0.053
SDNN (ms)	83 (66–102)	72 (59–111)	**0.553**	**0.011–0.846**	**0.026**
pNN50 (%)	27 (21–47)	27 (11–47)	0.256	−0.397–0.716	0.213
RMSSD (ms)	56 (44–83)	55 (33–73)	0.471	−0.136–0.815	0.060
**Frequency-domain indices**					
Total power (ms^2^)	6,012 (3,350–13,102)	4,604 (3,222–10,158)	**0.483**	**−0.063–0.814**	**0.045**
VLF power (ms^2^)	1,814 (1012–2797)	1,901 (1,006–2,393)	**0.634**	**0.290–0.833**	**0.001**
LF power (ms^2^)	935 (350–1688)	645 (391–1,377)	**0.720**	**0.270–0.911**	**0.004**
LF% (%)	40 (30–50)	46 (31–59)	**0.557**	**0.034–0.846**	**0.023**
HF power (ms^2^)	1,460 (677–2468)	789 (358–2,547)	0.335	−0.318–0.756	0.146
LF/HF ratio	0.67 (0.43–1.01)	1.047 (0.45–1.51)	**0.725**	**0.237–0.915**	**0.001**

Bold font indicates statistically significant differences (P < 0.05).

CI, confidence interval; HF, high frequency; HRV, Heart rate variability; ICC, intraclass correlation coefficient; LF, low frequency; LF%, normalized LF power; N-N, normal-to-normal; OSA, obstructive sleep apnea; pNN50, proportion of N-N50 divided by the total number of N-N intervals; RMSSD, square root of the mean of the sum of the squares of di?erences between adjacent N-N intervals; SDNN, standard deviation of all N-N intervals; VLF, very low frequency.

**Table 5:** Clinical variables, subjective quality and objective sleep quality of the study sample by altered OSA status in the outcome analysis.

**Variable**	**All participants**	**Stable residual OSA**	**Altered OSA**	***P* Value^a^**
***n***	**64**	**12**	**52**	
**Clinical variables**				
Age at diagnosis (years)	6 (5–9)	8 (6–10)	6 (5–8)	0.132
Male sex, *n* (%)	47 (73)	10 (83)	37 (79)	0.490
**BMI (kg/m** ^2^ **)**				
Preoperative	17.3 (15.0–21.9)	17.4 (15.3–23.0)	16.6 (14.7–21.9)	0.711
Postoperative	17.4 (15.1–22.8)	18.8 (16.4–24.5)	17.2 (15.0–22.2)	0.225
Change	4.0 (−0.4–1.4)	1.5 (−0.7–3.0)	0.4 (−0.4–1.1)	0.148
% Change	3 (−2–8)	7 (−4–16)	2 (−2–7)	0.135
**Systolic BP (mmHg)**				
Preoperative	103.5 (17.2)	105 (100–113)	103 (94–113)	0.558
Postoperative	105.3 (15.3)	106 (96–120)	102 (94–115)	0.642
Change	1.8 (14.9)	−1 (−9–10)	3 (−10–11)	0.783
% Change	3.2 (15.7)	−1 (−7–10)	3 (−9–11)	0.680
**Diastolic BP (mmHg)**				
Preoperative	65 (59–71)	61 (58–72)	65 (59–71)	0.444
Postoperative	64 (58–72)	64 (56–72)	64 (59–75)	0.530
Change	−1 (−7–7)	2 (−4–14)	−2 (−8–6)	0.136
% Change	−2 (−11–12)	4 (−6–25)	−3 (−12–9)	0.120
**Objective sleep quality (assessed by the OSA-18 questionnaire)**				
**OSA-18 score**				
Preoperative	81.8 (15.7)	89 (77–94)	82 (70–92)	0.404
Postoperative	52.0 (13.2)	54 (49–67)	50 (40–60)	0.127
Change	−26.4 (22.7)	−20 (−30–−6)	−32 (−43–−14)	0.072
% Change	−31 (24)	**−27 (−34–−3)**	**−39 (−48–−22)**	**0.037**
**Subjective sleep quality (assessed by polysomnography)**				
**OAHI (events/h)**				
Preoperative	5.8 (2.4–13.1)	5.0 (3.1–6.7)	7.7 (2.3–17.1)	0.318
Postoperative	1.4 (0.6–2.5)	**3.0 (2.4–6.4)**	**1.2 (0.5–1.8)**	**<** **0.001**
Change	−3.7 (−11.2–−1.2)	**−1.8 (−3.4–0.9)**	**−5.9 (−12.6–−1.6)**	**0.006**
% Change	−75 (−92–−45)	**−32 (−46–−20)**	**−87 (−94–−64)**	**<** **0.001**
**OAI (events/h)**				
Preoperative	0.6 (0.2–1.7)	0.4 (0.1–1.5)	0.4 (0–1.5)	0.931
Postoperative	0 (0–0.3)	**0.5 (0–0.8)**	**0 (0–0.2)**	**0.008**
Change	−0.3 (−1.0–0)	−0.2 (−0.9–0.3)	−0.3 (−1.1–0)	0.393
% Change	−79 (−100–0)	−55 (−98–95)	−85 (−100–0)	0.185
**Arousal index (events/h)**				
Preoperative	9.9 (7.3–16.9)	10.4 (8.0–15.5)	9.6 (7.2–17.7)	0.959
Postoperative	7.1 (6.0–9.2)	7.6 (6.0–9.0)	6.8 (6.0–9.8)	0.624
Change	−2.9 (−8.9–−3.3)	−2.2 (−7.3–−0.6)	−3.1 (−10.7–−0.3)	0.371
% Change	−33 (−53–−5)	−23 (−49–−6)	−34 (−53–−5)	0.667
**Mean SaO**_2_ **(%)**				
Preoperative	97 (97–98)	98 (97–98)	97 (97–98)	0.508
Postoperative	98 (97–98)	98 (97–98)	98 (97–98)	0.927
Change	0 (0–1)	0 (0–1)	0 (0–1)	0.731
% Change	0 (0–1)	0 (0–1)	0 (0–1)	0.589
**Minimal SaO**_2_ **(%)**				
Preoperative	90 (84–92)	90 (88–92)	90 (84–92)	0.904
Postoperative	92 (89–94)	91 (89–93)	92 (89–93)	0.316
Change	2 (−1–5)	1 (−2–6)	2 (0–5)	0.329
% Change	2 (−1–6)	1 (−2–6)	2 (0–6)	0.331
**N1 sleep (%)**				
Preoperative	10 (6–16)	11 (6–16)	10 (7–16)	0.918
Postoperative	9 (7–12)	7 (6–10)	9 (7–13)	0.212
Change	−2 (−9–2)	−4 (−8–1)	−2 (−9–3)	0.594
% Change	−24 (−51–41)	−30 (−55–23)	−20 (−50–52)	0.439
**N2 sleep (%)**				
Preoperative	39.2 (9.2)	**46 (40–54)**	**39 (31–44)**	**0.012**
Postoperative	41.6 (8.8)	47 (36–52)	41 (35–46)	0.232
Change	2.4 (10.5)	1 (−11–9)	4 (−2–10)	0.225
% Change	11.3 (32.3)	2 (−21–25)	10 (−8–32)	0.203
**N3 sleep (%)**				
Preoperative	27 (22–35)	25 (21–28)	28 (22–37)	0.081
Postoperative	25 (21–31)	27 (20–30)	25 (21–32)	0.810
Change	−1 (−8–5)	2 (−6–8)	−4 (−9–4)	0.180
% Change	3 (−30–24)	7 (−18–32)	−12 (−31–18)	0.235
**REM sleep (%)**				
Preoperative	19.1 (5.8)	19 (15–22)	21 (16–23)	0.636
Postoperative	22.4 (6.3)	22 (21–26)	22 (18–27)	0.925
Change	3.3 (7.4)	6 (−1–9)	3 (−3–8)	0.564
% Change	28.4 (53.0)	27 (−7–53)	14 (−14–56)	0.667
**TST (min)**				
Preoperative	337 (320–353)	338 (309–354)	337 (320–353)	> 0.999
Postoperative	336 (321–350)	335 (278–352)	337 (321–350)	0.763
Change	−6 (−32–21)	−8 (−50–15)	−4 (−32–23)	0.536
% Change	−2 (−9–6)	−3 (−13–5)	−1 (−9–8)	0.547

Data are expressed as median (interquartile range) or number (%). Bold font indicates statistically significant differences (P < 0.05).

BMI, body mass index; BP, blood pressure; OAHI, obstructive apnea-hypopnea index; OAI, obstructive apnea index; OSA, obstructive sleep apnea; REM, rapid eye movement; SaO2, blood oxygen saturation; TST, total sleep time.

**Table 6:** HRV indices of the study sample by altered OSA status in the outcome analysis.

**Variable**	**All participants**	**Stable residual OSA**	**Altered OSA**	***P* value^a^**
***n***	**64**	**12**	**52**	
**Time-domain indices**				
**N-N interval (ms)**				
Preoperative	793.7 (95.6)	803 (685–870)	786 (735–849)	0.945
Postoperative	827.0 (100.5)	829 (728–855)	828 (763–915)	0.390
Change	33.3 (108.8)	−25 (−112–107)	36 (−16–96)	0.216
% Change	5.1 (13.9)	−3 (−13–15)	5 (−2–12)	0.279
**SDNN (ms)**				
Preoperative	98.3 (34.1)	83 (66–102)	102 (75–125)	0.194
Postoperative	87.9 (29.6)	72 (59–111)	85 (69–110)	0.282
Change	−10.4 (30.1)	−5 (−29–9)	−13 (−29–13)	0.712
% Change	−4.8 (33.8)	−5 (−28–14)	−12 (−29–10)	0.606
**pNN50 (%)**				
Preoperative	37.1 (19.2)	27 (21–47)	38 (25–54	0.340
Postoperative	36.6 (21.5)	27 (11–47)	40 (20–53)	0.242
Change	−0.5 (19.0)	1 (−24–15)	−1 (−12–14)	0.612
% Change	28.7 (135.5)	5 (−55–86)	−2 (−30–39)	0.843
**RMSSD (ms)**				
Preoperative	63 (49–114)	56 (44–83)	70 (49–116)	0.371
Postoperative	61 (38–85)	55 (33–73)	64 (41–94)	0.249
Change	−9 (−30–10)	−2 (−36–13)	−10 (−28–8)	0.891
% Change	−9 (−32–22)	−1 (−40–38)	−10 (−32–17)	0.945
**Frequency-domain indices**				
**Total power (ms** ^2^ **)**				
Preoperative	9,454 (4,499–15,430)	6,012 (3,350–13,102)	9,505 (4,831–16,300)	0.169
Postoperative	6,437 (4,058–10,980)	4,605 (3,222–10,158)	6,739 (4,503–12,675)	0.180
Change	−2261 (−5568–1741)	−608 (−7189–1401)	−2,606 (−5,547–1,741)	0.904
% Change	−18 (−50–17)	−11 (−57–32)	−23 (−50–17)	0.655
**VLF power (ms** ^2^ **)**				
Preoperative	1,509 (1,095–2,727)	1,345 (771–2,718)	1,535 (1,157–2,727)	0.399
Postoperative	1,485 (1,032–2,102)	1,456 (852–3,691)	1,485 (1,063–2,102)	0.823
Change	92 (−734–540)	193 (−387–990)	42 (−779–497)	0.318
% Change	3 (−41–38)	19 (−30–56)	0 (−41–36)	0.460
**LF power (ms** ^2^ **)**				
Preoperative	1,243 (734–2,507)	935 (350–1,688)	1,286 (791–2,657)	0.144
Postoperative	942 (474–1,406)	645 (391–1,377)	1,022 (671–1,535)	0.279
Change	−155 (−924–231)	40 (−818–206)	−285 (−925–248)	0.439
% Change	−9 (−63–29)	9 (−56–96)	−20 (−63–19)	0.249
**LF% (%)**				
Preoperative	38 (29–48)	40 (30–50)	38 (28–48)	0.570
Postoperative	40 (32–50)	46 (31–59)	39 (32–49)	0.327
Change	2 (−8–13)	4 (−3–13)	2 (−10–13)	0.570
% Change	5 (−19–42)	10 (−13–33)	3 (−22–43)	0.823
**HF power (ms** ^2^ **)**				
Preoperative	1,970 (1,103–5,155)	1,460 (677–2,468)	2,234 (1,113–5,353)	0.122
Postoperative	1,364 (636–3,360)	789 (358–2547)	1,624 (812–3,420)	0.164
Change	−531 (−1,870–301)	−284 (−1601–282)	−663 (−2229–301)	0.536
% Change	−26 (−66–21)	−15 (−70–120)	−28 (−66–15)	0.559
**LF/HF ratio**				
Preoperative	0.62 (0.40–0.92)	0.67 (0.43–1.01)	0.61 (0.40–0.92)	0.570
Postoperative	0.70 (0.48–1.19)	1.05 (0.45–1.51)	0.66 (0.48–1.05)	0.294
Change	0.08 (−0.08–0.40)	0.16 (−0.07–0.49)	0.07 (−0.13–0.36)	0.310
% Change	13 (−12–70)	20 (−10–69)	13 (−14–72)	0.606

Data are expressed as median (interquartile range) or number (%).

^a^Data were compared using the Mann-Whitney U test or Fisher's exact test as appropriate.

HF, high frequency; HRV, Heart rate variability; LF, low frequency; LF%, normalized LF power; N-N, normal-to-normal; OSA, obstructive sleep apnea; pNN50, proportion of N-N50 divided by the total number of N-N intervals; RMSSD, square root of the mean of the sum of the squares of di?erences between adjacent N-N intervals; SDNN, standard deviation of all N-N intervals; VLF, very low frequency.

### 3.4. Associations between variables of interest at baseline

**Figure 3:**
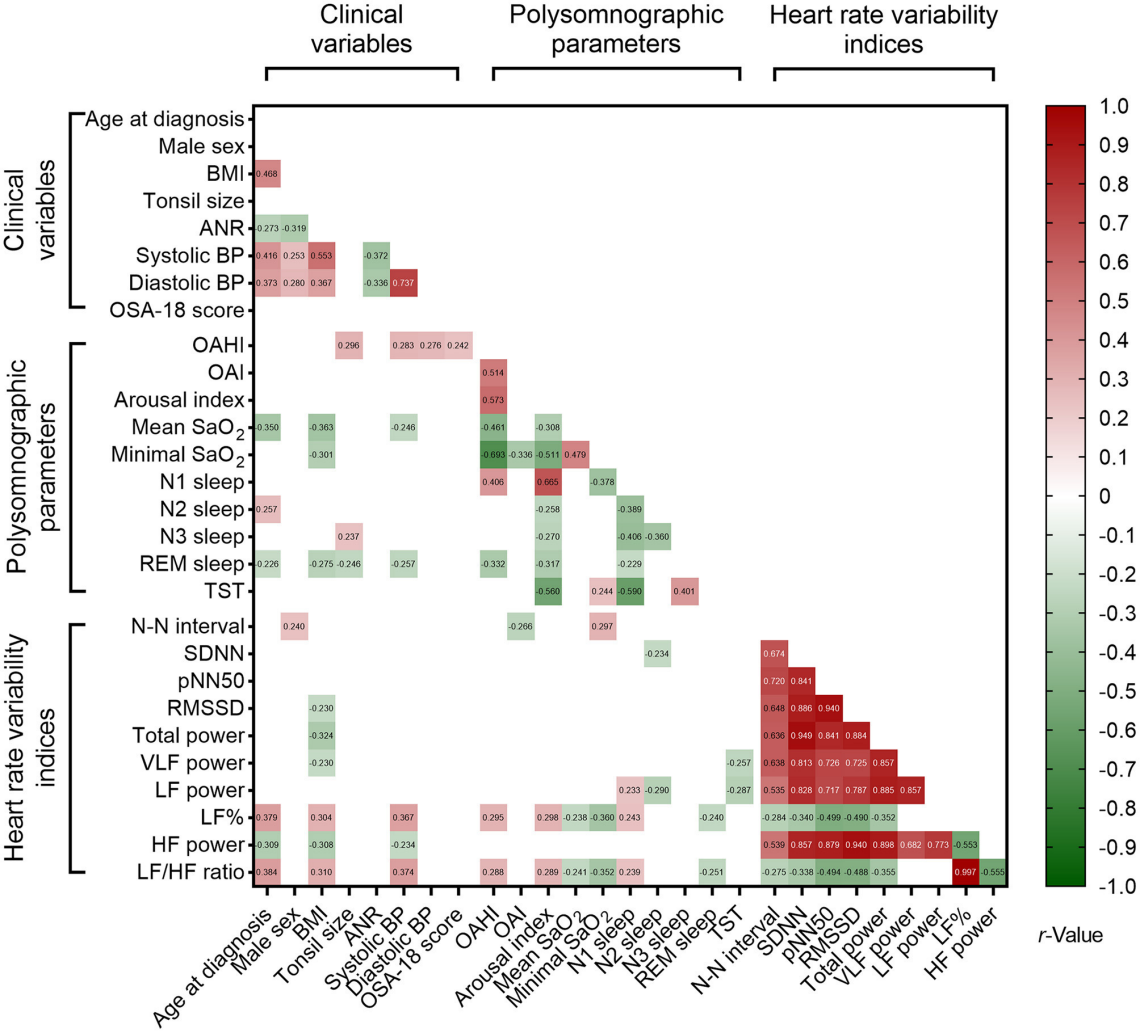
Associations of polysomnographic variables with clinical variables and sleep heart rate variability indices. Data are summarized as Pearson's or Point-Biserial rho, as appropriate. Blank spaces mean two-sided *P* ≥ 0.05.

Nested data structure and significant correlations were found among the polysomnographic parameters, several clinical variables and HRV indices (Figure 3). However, total OSA-18 questionnaire score was not associated with variables of interest. Using multivariable linear regression models (Table 7), male sex, OAHI and N3 sleep were independently associated with tonsil size, and systolic BP, OAHI and VLF power were independently associated with ANR. Furthermore, tonsil size, diastolic BP and LF% were independently correlated with OAHI. Table 7 summarizes the independent associations of other polysomnographic parameters with the variables of interest.

**Table 7:** Multivariable linear regression models of independent associations of tonsil/adenoid sizes and polysomnographic parameters with other variables in the baseline analysis.

**Baseline variables**	**Independent variables**	**β (95% CI)**	**VIF**	***P* value**	**Adjusted *R*^2^**
**Clinical variables**					
Tonsil size	Male sex	−0.33 (-0.60-−0.06)	1.00	0.019	0.216
	OAHI	0.01 (0.001–0.02)	1.01	0.027	
	N3 sleep	0.02 (0.01–0.04)	1.01	0.005	
ANR	Systolic BP	−0.003 (-0.005-−0.002)	1.14	< 0.001	
	OAHI	0.003 (0.001–0.005)	1.16	0.005	
	VLF power	−0.00001 (-0.00003-−0.000003)	1.02	0.018	
**Subjective sleep quality (polysomnographic parameters)**					
OAHI	Tonsil size	7.96 (3.03–12.89)	1.02	0.002	0.243
	Diastolic BP	0.29 (0.02–0.56)	1.04	0.034	
	LF%	0.25 (0.07–0.44)	1.06	0.008	
OAI	OSA-18 score	0.07 (0.001–0.14)	1.00	0.049	0.119
	N-N interval	−0.01 (-0.03-−0.003)	1.00	0.015	
Arousal index	N-N interval	−0.05 (-0.08-−0.02)	1.46	0.001	0.283
	LF power	0.01 (0.003–0.01)	2.72	< 0.001	
	HF power	−0.001 (-0.003-−0.0002)	2.71	0.020	
Mean SaO_2_	BMI	−0.10 (-0.15-−0.05)	1.10	< 0.001	0.228
	N-N interval	0.004 (0.001–0.01)	1.75	0.001	
	VLF power	−0.0002 (-0.0004-−0.0001)	1.84	0.007	
Minimal SaO_2_	BMI	−0.50 (-0.74-−0.25)	1.09	< 0.001	0.319
	N-N interval	0.04 (0.02–0.06)	1.75	< 0.001	
	VLF power	−0.001 (-0.002-−0.001)	1.83	< 0.001	
N1 sleep	pNN50	−0.22 (-0.38-−0.06)	2.08	0.007	0.146
	LF power	0.004 (0.002–0.01)	2.08	0.001	
N2 sleep	LF power	−0.002 (-0.003-−0.0004)	1.00	0.012	0.072
N3 sleep	Tonsil size	3.59 (0.04–7.14)	1.00	0.048	0.123
	pNN50	0.15 (0.01–0.29)	2.13	0.038	
	VLF power	−0.001 (-0.003-−0.0001)	2.12	0.030	
REM sleep	Age	−0.68 (-1.30-−0.05)	1.00	0.036	0.116
	Tonsil size	−2.88 (-5.38-−0.39)	1.00	0.027	
TST	LF power	−0.01 (-0.01-−0.002)	1.00	0.013	0.070

ANR, adenoidal-nasopharyngeal ratio; BMI, body mass index; BP, blood pressure; CI, confidence interval; HF, high frequency; LF, low frequency; OAHI, obstructive apnea-hypopnea index; OAI, obstructive apnea index; OSA, obstructive sleep apnea; N-N, normal-to-normal; pNN50, proportion of N-N50 divided by the total number of N-N intervals; REM, rapid eye movement; SaO_2_, blood oxygen saturation; TST, total sleep time; VIF, variance inflation factor; VLF, very low frequency.

### 3.5. Changes in the variables of interest after adenotonsillectomy

The median follow-up period was 4 (IQR, 3–6) months. In outcome analysis, mean SaO_2_, minimal SaO_2_ and rapid eye movement sleep significantly increased, and OSA-18 score, OAHI, OAI, arousal index and N1 sleep significantly reduced after adenotonsillectomy (Table 5).

Regarding HRV indices, SDNN, total power and HF power significantly reduced after adenotonsillectomy (Table 6), and they were still significantly different from normal values (47) (Figure 2).

### 3.6. Associations of percentage changes in the variables of interest

**Figure 4:**
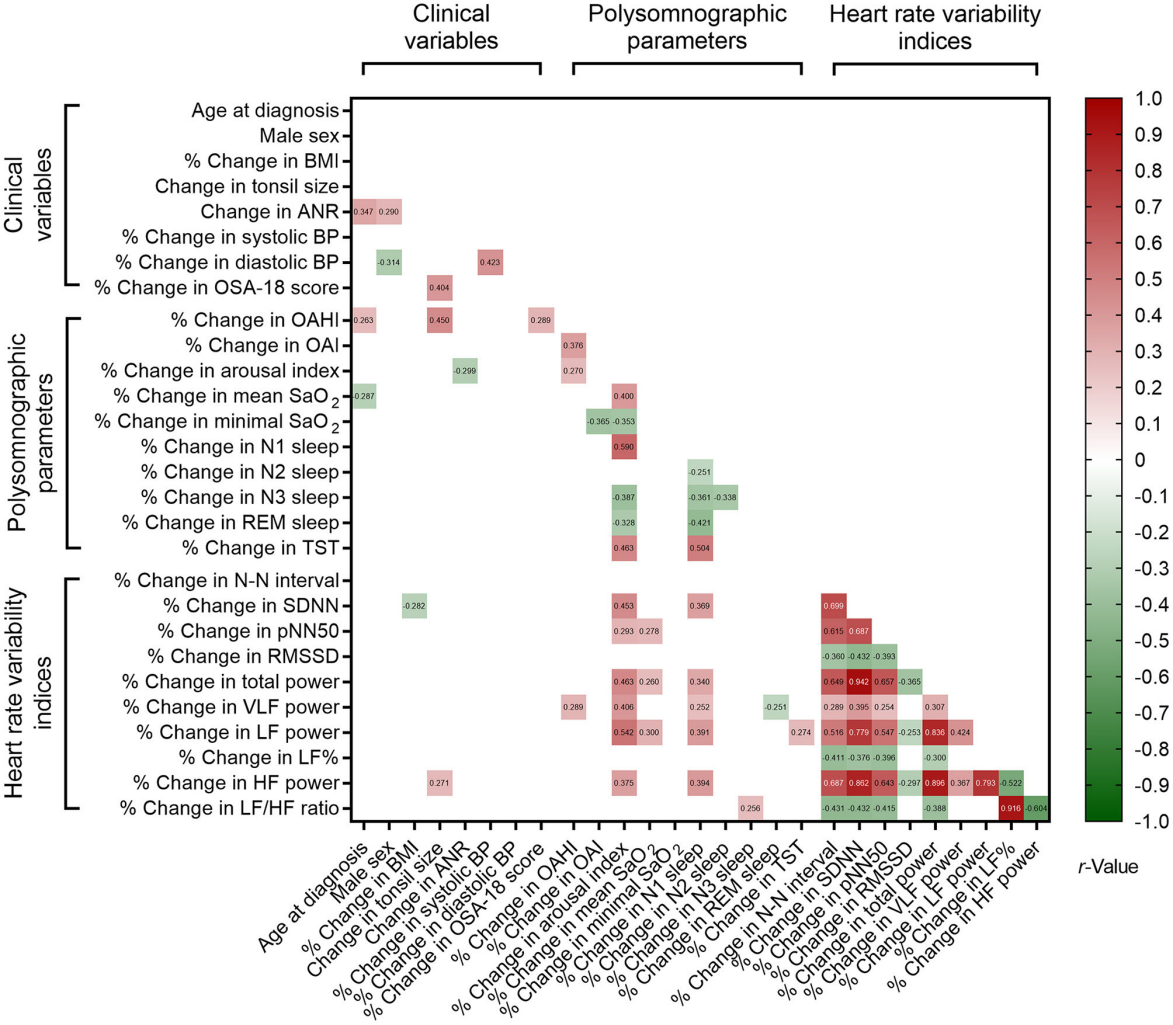
Associations of % changes in polysomnographic variables, % changes in clinical variables, and % changes in sleep heart rate variability indices. Blank spaces mean two-sided *P* ≥ 0.05.

Correlations of % changes in polysomnographic parameters and % changes in clinical variables and HRV indices also revealed significant associations with nested data structure (Figure 4). Using multivariable linear regression models (Table 8), % changes in OSA-18 score, OAHI and HF power were independently associated with change in tonsil size. Age at diagnosis, male sex and % change in arousal index were independently associated with change in ANR, and change in tonsil size was independently correlated with % change in OAHI.

**Table 8:** Multivariable linear regression models of independent associations of changes in tonsil/adenoid size and % changes in polysomnographic parameters in the outcome analysis.

**Outcome variables**	**Independent variables**	**β (95% CI)**	**VIF**	***P* Value**	**Adjusted *R*^2^**
**Clinical variables**					
Change in tonsil size	% Change in OSA-18 score	0.01 (0.002–0.01)	1.10	0.005	0.309
	% Change in OAHI	0.002 (0.001–0.004)	1.11	0.005	
	HF power	0.001 (0.0001–0.002)	1.03	0.025	
Change in ANR	Age at diagnosis	0.01 (0.001–0.03)	1.06	0.030	0.203
	Male sex	0.06 (0.004–0.12)	1.04	0.037	
	% Change in arousal index	−0.0002 (-0.0004-−0.0003)	1.02	0.026	
**Subjective sleep quality (polysomnographic parameters)**					
% Change in OAHI	Change in tonsil size	65.79 (32.37–99.20)	1.00	< 0.001	0.189
% Change in OAI	None				
% Change in arousal index	Change in ANR	−317.27 (-593.74-−40.794)	1.02	0.025	0.329
	% Change in LF power	0.84 (0.49–1.18)	1.02	< 0.001	
% Change in mean SaO_2_	Age at diagnosis	−0.17 (-0.31-−0.03)	1.05	0.020	0.296
	% Change in N-N interval	−0.05 (-0.08-−0.03)	1.74	< 0.001	
	% Change in pNN50	0.004 (0.002–0.01)	1.78	0.002	
	% Change in LF power	0.004 (0.001–0.01)	1.55	0.024	
% Change in minimal SaO_2_	None				
% Change in N1 sleep	% Change in LF power	0.55 (0.21–0.88)	1.00	0.002	0.138
% Change in N2 sleep	None				
% Change in N3 sleep	% Change in LF/HF ratio	0.09 (0.002–0.18)	1.00	0.045	0.050
% Change in REM sleep	% Change in VLF power	−0.17 (-0.34-−0.004)	1.00	0.045	0.048
% Change in TST	% Change in LF power	0.17 (0.02–0.32)	1.00	0.031	0.060

BMI, body mass index; CI, confidence interval; HF, high frequency; LF, low frequency; OAHI, obstructive apnea-hypopnea index; OAI, obstructive apnea index; OSA, obstructive sleep apnea; N-N, normal-to-normal; pNN50, proportion of N-N50 divided by the total number of N-N intervals; REM, rapid eye movement; SaO2, blood oxygen saturation; TST, total sleep time; VIF, variance inflation factor; VLF, very low frequency.

### 3.7. Mediation and moderation analyses

**Figure 5:**
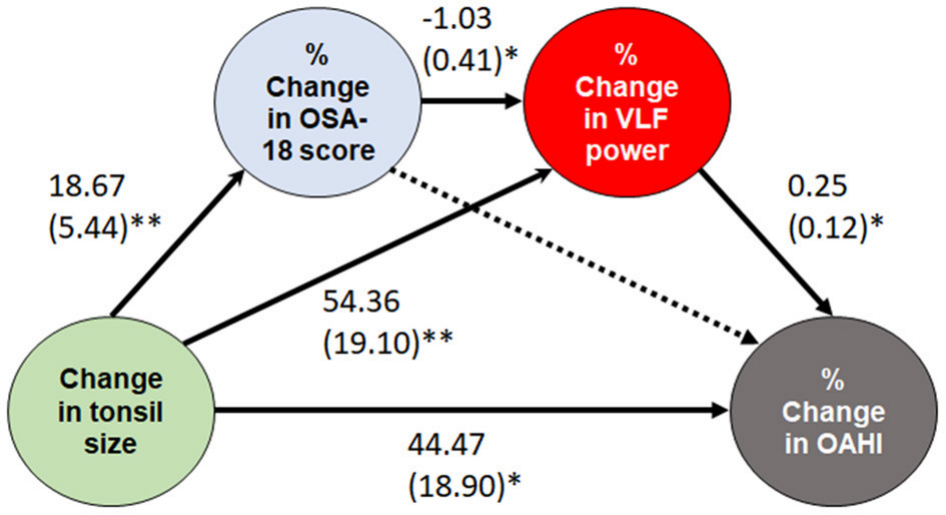
A serial multiple mediation model of the effect of change in tonsil size. Data are summarized as β and standard errors. *P < 0.05 and ≥ 0.01; **P < 0.01 and ≥ 0.001. Solid lines indicate significant paths, while a dotted line indicates a non-significant path. OAHI, obstructive apnea-hypopnea index; OSA, obstructive sleep apnea; VLF, very low frequency.

Consistent relationships between “tonsil size and OAHI” and “change in tonsil size and % change in OAHI” were observed. Mediation and moderation analyses were performed from change in tonsil size to % change in OAHI, especially with regards to HRV indices, and only a significant conceptual serial multiple mediation model was identified: change in tonsil size (independent variable), % change in OSA-18 score (first mediator), % change in VLF power (second mediator), and % change in OAHI (dependent mediator) (Figure 5). The direct paths from change in tonsil size to % change in OAHI, change in tonsil size to % change in OSA-18 score, change in tonsil size to % change in VLF power, change in OSA-18 to % change in VLF power, and % change in VLF power to % change in OAHI were significant. In contrast, the direct paths from change in OSA-18 to % change in OAHI were not significant. The serial mediation model revealed a positive total effect (β = 65.78, standard error = 16.71, *P* < 0.001). The direct effect of change in tonsil size on % change in OAHI (β = 44.47, standard error = 18.90, *P* = 0.022) was significant. For the indirect effects, the first path from change in tonsil size to % change in OAHI through % change in OAS-18 score (effect = 12.44, 95% CI:−5.18–32.99) was not significant. The second path through % change in VLF power (effect = 13.74, 95% CI: 0.01–33.36), third path through % change in OAS-18 score and % change in VLF power (effect = −4.87, 95% CI:−13.69-−0.09), and indirect effect (effect = 21.32, 95% CI: 0.39–44.30) were significant.

## 4. Discussion

This study is the first to report that OSA-related quality of life and VLF power were first and second mediators of the relationship between tonsil size and improvement in AHI using a conceptual serial multiple mediation model. Beyond providing important mechanistic insights, these results suggest that VLF power could be a new target for OSA therapy in children. For example, exercise training can decrease VLF power over time (49) and reduce AHI (50) in adults.

Our results confirmed the reproducibility of sleep HRV measurements at two time points. Most measures showed moderate or substantial reproducibility, except for pNN50 and HF power. The possible reason for this relatively lower reproducibility may be related to sleep stage and arousal index. To the best of our knowledge, no comprehensive reproducibility study has reported HRV measurements in children with OSA. Accordingly, the interpretations of sleep pNN50 and HF power should be made with caution in this population.

Using full-night HRV measurements, SDNN, total power, and VLF power in the children with OSA were significantly higher than normal values (Figure 2) (14, 47, 48). SDNN and total power represent total capacity of the regulation system, whilst VLF power represents sympathetic activity (36) (Table 1). Although SNS and PNS activities both contribute to SDNN and total power, long-term recordings have revealed that SNS activity is more related to these indices (51). The transition between normal and pathological respiration can enhance SNS activity rather than PNS activity in adults with OSA (52). Additionally, the results of this and previous studies (13, 14, 53) suggest that sympathetic activity increases during sleep in children with OSA; however, SDNN in the 12 obese children with OSA in this study was comparable to normal values (47) (Table 3). This discrepancy may be explained by the patients' weight status, since childhood obesity is significantly related to low SDNN (54). Furthermore, this study and Isaiah's study (14) found that % changes in SDNN and total power were not related to % change in OAHI. Therefore, these changes could not be simply due to improvements in OAHI after adenotonsillectomy.

The baseline values and % changes in tonsil size and ANR were not consistently associated with most HRV indices. Despite increased ANR being related to decreased VLF power in children with OSA, the causal relationship between adenoid hypertrophy and reduced sympathetic activity could not be supported by the post-operative changes. Nevertheless, our findings suggested a positive relationship between change in tonsil size and % change in HF power of HRV (parasympathetic modulation). We hypothesize that tonsillectomy may directly injure or cause scar formation, thereby reducing function of the lingual branch of the hypoglossal nerve, interrupting baroreceptor signaling at the carotid sinus, influencing vagus nerve function, eventually resulting in decreased parasympathetic modulation and increased sympathetic activity of cardiac autonomic function during sleep. This condition may further interfere with the relationships between % change in SDNN or total power and % change in OAHI.

Our results demonstrated significant relationships between the change in tonsil size and % change in OAHI as well as relationships between the change in tonsil size and % change in OSA-18 score as previous studies (29, 55). Tonsil size has been significantly associated with the change in OSA-18 score after tonsillectomy in children with sleep-disordered breathing (56). Although a change in AHI has been associated with a change in OSA-18 score (23), we found that this association was not independent in this study. In simple mediation and moderation models, % change in OSA-18 score neither mediated nor moderated the relationship between the change in tonsil size and % change in OAHI. However, in serial mediation analysis, the relationship between the change in tonsil size and % change in OAHI was mediated by % change in OSA-18 score and % change in VLF power in serial analysis, and also by % change in VLF power alone (Figure 5).

VLF rhythm is a cardiac intrinsic rhythm which is essential for health and happiness (36). Even though there is currently no agreement on the physiological mechanisms responsible for activity within the VLF band, low VLF power has been associated with adverse outcomes and all-cause mortality (57, 58). This band is generated by the stimulation of afferent sensory neurons in the heart (59). In animal models, stressful stimulation (60) and paradoxical sleep deprivation (61) have been shown to significantly reduce VLF power (60). In this study, the inverse relationship between % change in OSA-18 and % change in VLF power suggested that reduced OSA-specific stress and sleep disturbance may increase sleep VLF power.

However, VLF power is an independent predictor of AHI in humans (62). VLF power is significantly elevated during pathological respiration compared with normal respiration in OSA patients (52). Therefore, it is reasonable that reduced AHI may contribute to a decrease in VLF power after adenotonsillectomy. Our mediation model also highlighted the possibility that changes in VLF power may influence changes in AHI in children with OSA. Sympathetic abnormalities were shown to precede the development of mild OSA in a cohort of adults with no known diagnosis of OSA (63). Although further direct evidence is warranted, these studies suggest that a reduction in VLF power may help to alleviate the AHI in children with OSA.

Therefore, the mediation role of VLF power on the relationship between change in tonsil size and % change in AHI is of interest. Increasing exercise intensity can reduce awake VLF power (49), and morning exercise can increase sleep VLF power (64) in adults. Besides, exercise training (65) or aerobic exercise combined with resistance training can reduce AHI in adults. Therefore, VLF power is modifiable and may be a marker of therapeutic efficacy and a potential therapeutic target for OSA (66). However, in children with adenotonsillar hypertrophy, it may be unlikely that addressing the HRV independently will improve AHI unless there is a clear demonstration the neuromuscular tone is improved to the point that the tonsils do not medialize during sleep. Accordingly, future studies should focus on VLF power-lowering interventions and their effects on the severity of childhood OSA.

### 4.1. Strengths and limitations

Compared with previous studies (13, 14, 17, 48), the greatest strengths of this investigation were the inclusion of a sample of representative and well-characterized pediatric OSA patients. Our results provided a preliminary yet comprehensive documentation of the relationships of HRV indices with clinical variables and polysomnographic parameters before and after adenotonsillectomy, which showed some novel and interesting findings. However, limitations should be addressed. First, the HRV results may have been affected by certain psychophysiological changes (e.g., anthropometrics, lifestyle factors, acute or chronic diseases) other than adenotonsillectomy. However, the use of standardized, full-night, in-laboratory protocols with moderate-to-substantial reproducibility in most HRV indices among OSA patients with stable severity reduces this concern. Second, approximately half of our subjects received both adenotonsillectomy and medical treatment, and the heterogeneity of care may have had a confounding effect. Nevertheless, these interdisciplinary treatments are closer to real-world care for OSA, and a greater variability in AHI changes may contribute to better generalizability of this study. Third, 3 months may not be long enough to show cardiovascular changes, and studies with a longer follow-up period are warranted for this young population. Finally, in this study, there was no evidence of direct mediations of HRV on the relationship between adenotonsillectomy and AHI or AHI on the relationship between adenotonsillectomy and HRV indices (14), and this may be due to difficulties in measuring HRV across various sleep stages. Among school-age children, excessive body movements and parasomnia (67) make the researchers need 2-min epochs to analyze HRV and choose sleep periods free of respiratory events and movement artifacts (17). However, frequency-domain measurements, such as VLF power and LF/HF ratio, often require recording periods of at least 5 min (34). Therefore, measuring HRV across various stages in our study population is challenging. Nevertheless, averages may not be sensitive enough to detect sleep stage-specific mediating effects. In future studies, ultra-short-term HRV measurements during different sleep stages should be conducted to accurately assess the impact of nocturnal HRV changes on the management of OSA.

## 5. Conclusion

In conclusion, we confirmed that analysis of electrocardiographic polysomnography signals is a reliable method to measure HRV over 3 months in children with OSA. Adenotonsillectomy either reduced AHI or sympathetic activity during sleep. Improved OSA-specific quality of life and reduced sleep VLF power serially mediated the relationship between the change in tonsil size and % change in AHI. These findings suggest that HRV measurement may help monitor the sleep quality status and the disease burden of childhood OSA and many other venues. Our preliminary results also support applications of wireless HRV measurements with high-fidelity psychophysiology acquisition using edge computing in the patient's natural sleeping environment to overcome the highly obtrusive effects of visiting the sleep laboratory (68). This technology can potentially be a “platinum standard” of sleep studies instead of the traditional “gold standard” of in-laboratory polysomnography.

## Data availability statement

The original contributions presented in the study are included in the article/supplementary material, further inquiries can be directed to the corresponding authors.

## Ethics statement

The studies involving human participants were reviewed and approved by Institutional Review Board of Chang Gung Medical Foundation, Taoyuan, Taiwan. Written informed consent for participation was not provided by the participants' legal guardians/next of kin because: the current study was based on a secondary analysis of existing data. We provided a copy of the approval by the Institutional Review Board of Chang Gung Medical Foundation (No. 202200882B0), which approved the waiver of the participants' consent.

## Author contributions

L-AL, H-HC, TK, and CY conceptualized and designed the research project. L-AL, H-HC, H-SH, C-YW, TK, and CY interpreted the data. L-AL, H-HC, and H-SH collected the data and wrote the initial manuscript. C-YW, L-PC, H-YL, T-JF, Y-SH, G-SL, AY, TK, and CY contributed to writing and editing the manuscript. All authors have read and agreed to the published version of the manuscript.
